# Chemopreventive and Therapeutic Effects of Edible Berries: A Focus on Colon Cancer Prevention and Treatment

**DOI:** 10.3390/molecules21020169

**Published:** 2016-01-30

**Authors:** Sadia Afrin, Francesca Giampieri, Massimiliano Gasparrini, Tamara Y. Forbes-Hernandez, Alfonso Varela-López, José L. Quiles, Bruno Mezzetti, Maurizio Battino

**Affiliations:** 1 Dipartimento di Scienze Cliniche Specialistiche ed Odontostomatologiche (DISCO)-Sez. Biochimica, Facoltà di Medicina, Università Politecnica delle Marche, Ancona 60131, Italy; dolla.bihs@gmail.com (S.A.); f.giampieri@univpm.it (F.G.); m.gasparrini@univpm.it (M.G.); tamara.forbe@gmail.com (T.Y.F.-H.); 2 Department of Physiology, Institute of Nutrition and Food Technology ‘‘José Mataix”, Biomedical Research Centre, University of Granada, Armilla, Avda. del Conocimiento s.n., Armilla 18100, Spain; avarelalopez@gmail.com (A.V.-L.); jlquiles@ugr.es (J.L.Q.); 3 Dipartimento di Scienze Agrarie, Alimentari e Ambientali, Università Politecnica delle Marche, Via Ranieri 65, Ancona 60131, Italy; b.mezzetti@univpm.it; 4 Centre for Nutrition & Health, Universidad Europea del Atlantico (UEA), Santander 39011, Spain

**Keywords:** berry, chemoprevention, colon cancer, polyphenol, bioactive compound

## Abstract

Colon cancer is one of the most prevalent diseases across the world. Numerous epidemiological studies indicate that diets rich in fruit, such as berries, provide significant health benefits against several types of cancer, including colon cancer. The anticancer activities of berries are attributed to their high content of phytochemicals and to their relevant antioxidant properties. *In vitro* and *in vivo* studies have demonstrated that berries and their bioactive components exert therapeutic and preventive effects against colon cancer by the suppression of inflammation, oxidative stress, proliferation and angiogenesis, through the modulation of multiple signaling pathways such as NF-κB, Wnt/β-catenin, PI3K/AKT/PKB/mTOR, and ERK/MAPK. Based on the exciting outcomes of preclinical studies, a few berries have advanced to the clinical phase. A limited number of human studies have shown that consumption of berries can prevent colorectal cancer, especially in patients at high risk (familial adenopolyposis or aberrant crypt foci, and inflammatory bowel diseases). In this review, we aim to highlight the findings of berries and their bioactive compounds in colon cancer from *in vitro* and *in vivo* studies, both on animals and humans. Thus, this review could be a useful step towards the next phase of berry research in colon cancer.

## 1. Introduction

In the United States colon cancer is the second most prevalent cause of death from cancer in men and women after lung cancer; in 2014, an estimated 96,830 new cases of colon cancer and 50,310 patient deaths were reported [1]. In Europe, colorectal cancer (CRC) is the second most common cancer, with almost 500,000 new cases diagnosed in 2012 [2], while over 1 million new cases are diagnosed each year worldwide [3].

Multiple factors are associated with the development of CRC, including high alcohol consumption (60% greater risk) [4], high-fat diet poor in fiber, red meat, obesity, smoking (20% associated with CRC), lack of physical exercise [5], diabetes [6], older age [7], inflammatory bowel disease (ulcerative colitis and Crohn’s disease) [8], family history (20% cases of CRC) [9] and some genetic syndromes [hereditary nonpolyposis colorectal cancer and familial adenomatous polyposis (FAP)] [10].

Several genetic and epigenetic alterations may increase the incidence of sporadic colon cancer through distinct molecular mechanisms. Microsatellite instability, chromosomal instability, and CpG island methylator phenotype are the main pathways in CRC pathogenesis [11]. Mutations in adenomatous polyposis coli (APC) gene can lead to the activation of the wingless-type (Wnt) pathway, a common mechanism for initiating polyp to cancer progression sequence [12]. *p53* is a tumor suppressor (transcriptional factor) that controls cell cycle, apoptosis and DNA repair mechanisms. Mutation of this gene is one of the familiar genetic changes in the development of CRC [13]. In addition, mutations of oncogenes, Kirsten rat sarcoma virus oncogene homolog or B-raf proto-oncogene, occur in approximately 55% to 60% of CRCs, aberrantly activating the mitogen-activated protein kinase (MAPK) signaling pathway [14,15]. In CRC, epidermal growth factor receptor is also expressed in 60% to 80% of cases [16], and has been associated with multiple signaling pathways such as RAS-RAF-MEK-MAPKs and PI3K/Akt [17]. Mutations in the transforming growth factor β (TGF-β) signaling pathway may also be involved in the progression of cancer. Mutations in type II TGF-β receptor gene occur in around 30% of CRCs [18,19,20]. Furthermore, an aberrant nuclear factor-kappa B (NF-κB) activation has been detected in more than 50% of colorectal and colitis-associated tumors [21].

Colorectal cancer prevention usually depends on screening methods, including stool tests, radiographic imaging and colonoscopy to identify adenomatous polyps, a precursor lesion for colon cancer. Treatments used for colorectal cancer may include some combination of surgery, radiation therapy, chemotherapy and targeted therapy. In spite of the advances in colon cancer treatment, including postoperative care, recurrence and mortality rates remain high; hence the urgent need to complement the current therapies. Depending on the cancer stage and patient’s features, several chemotherapy drugs and regimens for CRC management are proposed. The use of various 5-fluorouracil (5-FU) based chemotherapeutics as neoadjuvants such as FOLFOX and FOLFIRI, along with bevacizumab, panitumumab, or cetuximab, depends on the individual patient and tumor characteristics [22].

Besides the limitations of current cancer management (surgery, chemotherapy and radiotherapy), available cytotoxic drugs are not easily affordable or available in certain places (especially in developing countries), and their use is also associated with a number of undesirable side and adverse effects [23]. As a consequence, a large proportion of the population prefers to patronize complementary and alternative medicine (CAM) [24]. Despite its own limitations, CAM has a number of advantages (such as affordability, availability and lower side effects) compared to synthetic or standard drugs [25]. The use of various natural and synthetic drugs for CRC prevention has indeed attained remarkable attention in recent years, [26]; in this context, fruit and vegetables including soft fruits such as berries may represent a valid alternative, because of their chemopreventive or chemotherapeutic properties against certain diseases, such as cancer [27]. It is known that 10%–70% of all cancers is correlated with diet and about 90% of colorectal cancer may be preventable through alterations of diet [28], since dietary deficiencies may alter sensitivity to genetic damage and influence carcinogen metabolism contributing to colon cancer development [29].

**Figure 1:**
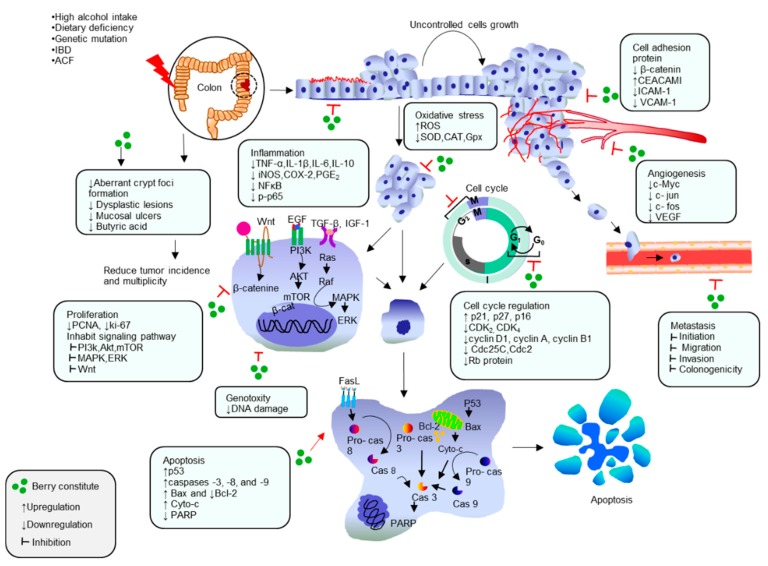
Berries exhibit chemopreventive and therapeutic response against colon cancer by targeting on cellular functions and signal transduction pathways associated with anti-inflammatory, antioxidative, antiproliferative, cell cycle regulation, apoptotic, antiangiogenesis and anti-metastasis mechanism.

Berries are a common functional fruit worldwide and are among the richest fruits in natural compounds, including minerals, vitamins, dietary fibers, and especially polyphenolic phytochemicals [30,31,32]. In recent decades, polyphenolic compounds of berries have attracted substantial attention and have been subjected to extensive research due to their antioxidant properties, potential in health promotion and disease prevention, thus improving safety and consumer acceptability [33,34,35,36]. Therefore, in this review we highlight the latest developments on the preventive and therapeutic activities of berries and their bioactive compounds from *in vitro* and *in vivo* studies on animal and humans, against colon cancer. Particularly, we discuss their molecular activities, such as: (i) protection of cells from oxidative damage; (ii) suppression of inflammation; (iii) inhibition of cell proliferation by regulation of cell cycle and induction of apoptosis; (iv) protection and reconstruction of DNA damage, as well as (v) inhibition of angiogenesis (Figure 1).

## 2. Bioactive Compound Profile and Antioxidant Capacity of Berries

The interesting profile of berries has been reported by non-nutritive, nutritive and phytochemical assessments. Extraction (water, methanol, ethanol, chloroform, hexane, ethyl acetate and acetone) and structural elucidation (HPLC, nuclear magnetic resonance and mass spectrometry) have identified many components and their relevant abundance. Berries are rich in various bioactive compounds including phenolic acids, benzoic acid (hydroxybenzoic) and derivatives of cinnamic acid (hydroxycinnamic acids) [37], stilbenes (resveratrol) [38], lignans (secoisolariciresinol) [39], flavonoids including anthocyanins (cyanidin, pelargonidin, delphinidin, peonidin, malvidin) [40], flavonols (quercetin, myricetin, kaempferol) and flavanols (catechins) [41], condensed tannins (proanthocyanidins) [42] and hydrolyzable tannins (ellagitannins and gallotannins) [43], vitamins (vitamins A, C, E) [44,45], folate [46], alkaloids (berberine, berbamine and palmatine) [47], carotenoids [48], xanthones (α-mangostin, β-mangostin, γ-mangostin, and methoxy-β-mangostin) [49] and polysaccharide [50]. Major bioactive compounds found in different berries are presented in Table 1.

Diversity of berry phenolics is observed in several ways, including: (i) genetic and environmental factors, such as species and variety, cultivation methods, fertilization, weather, ripeness and harvesting season, conditions and time of storage [51,52,53,54,55,56,57]; (ii) chemical structures, ranging from simple single-aromatic ring compounds to large complex molecules built up from multiple smaller ones [58]; (iii) degree of oxidation and substitution patterns of hydroxylation; (iv) abilities to exist as stereoisomers; (v) glycosylation by sugar moieties and other substituents; and (vi) conjugation to form polymeric molecules [59].

**Table 1:** Major bioactive compounds present in different berries.

Berries	Major Bioactive Compounds						
	Flavonoids	Phenolic Acids	Tannins	Vitamins	Stilbenes	Other Compounds	References
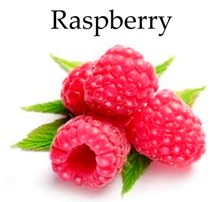	Anthocyanins (Cyanidin glycosides, cyaniding-3-arabinose, cyanidin-3-soporoside, Cyanidin-3-rutinoside, and pelargonidin glycosides), quercitin, catechin, epicatechin, apigenin, chrysin and naringenin	Caffeic acid, ferulic acid, gallic acid, chlorogenic acid, *p*-coumaric acid and *p*-hydroxybenzoic acid	Ellagitannin and ellagic acid	Folate, Vitamin C and B	Resveratrol	Polyunsaturated fatty acids, calcium, potassium, magnesium, phosphorus, lutein, α and β carotene	[60,61]
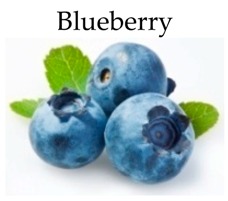	Anthocyanins (malvidin glycosides, cyanidin glycosides, delphinidin glycosides and petunidin glycosides), myricetin glycosides, quercetin glycosides, kaempferol, (+)-catechin and (−)-epicatechin	Benzoic and cinnamic acids	Proanthocyanidins	Vitamin C, B complex, E, A and ascorbic acid	Pterostilbene	Potassium, calcium, magnesium, phosphorus, β-carotene and lutein	[62,63]
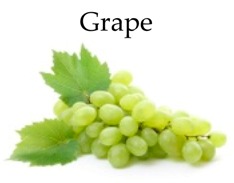	Anthocyanins (malvidin-3-glucoside, peonidin-3-glucoside, cyanidin-3-glucoside and petunidin-3-glucoside), quercetin, kaempferol, (+)-catechin, epicatechin and epicatechin gallate	Hydroxycinnamic acid, gallic acid, caffeic acid, coumaric acid and ferulic acid	Proanthocyanidins and ellagic acid	Vitamin C and K	Resveratrol, pterostilbene, piceid, viniferins, astringin and piceatannol	Copper, carotenoids (β-carotene and lutein), and melatonin	[64,65]
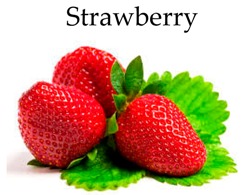	Anthocyanins (cyanidin-3-glucoside, pelargonidin and pelargonidin-3-rutinoside), quercetin glycosides, kaempferol glycosides and flavan-3-ols ((+)-catechin)	Hydroxycinnamic acids, gallic acid, caffeic acid, *p*-coumaric acid and coumaroyl glycosides	Proanthocyanidins, ellagitannins, gallotannins, ellagic acid and its glycosides.	Folate and Vitamin C	Resveratrol	Potassium, calcium, magnesium and phosphorus	[66,67]
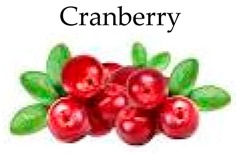	Anthocyanins(cyanidin glycosides, peonidin glycosides, pelargonidin glycosides, malvidin glycosides, delphinidin glycosides) kaempferol and quercetin	*p*-Coumaric acid and hydroxycinnamic acid	Proanthocyanidins	Folate, Vitamin C and A	Resveratrol	Calcium, iron, potassium, magnesium and mamganese	[61,68]
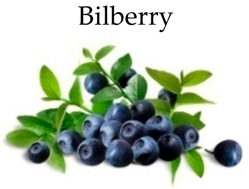	Anthocyanin (cyanidin-3-galactoside, cyanidin-3-glucoside and cyanidin-3-arabinoside) , quercetin	Chlorogenic acid, Caffeic acid derivative	Proanthocyanidins	Ascorbic acid	Resveratrol	Carotenoids, sterols and lipids	[69,70]
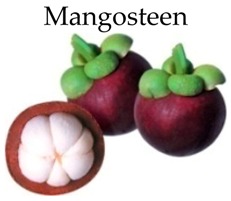		p-Hydroxybenzoic acid, m-hydroxybenzoic and 3,4-dihydroxy-mandelic	Proanthocyanidins	Folate		α-Mangostin, β-Mangostin, µ-Mangostin, 1,3,6,7-Tetrahydroxy Xanthone, 1-Isomangostin, Mangosharin, calcium, potassiuum and magnesium	[71,72,73,74]
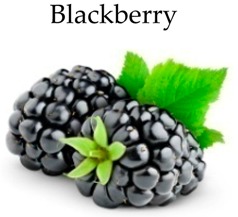	Anthocyanins (cyanidin glycosides, pelargonidin glycosides, peonidin glycosides), quercitin, cyaniding and epicatechin	Gentisic acid, protocatchiuic acid, salicylic acid and caffeic acid	Ellagitannins and ellagic acid	Folate and Vitamin C (ascorbic acid)		β-carotene, cryptoxanthin and lutein	[60]
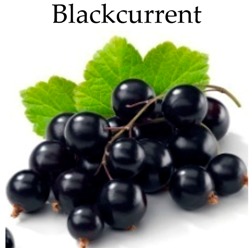	Anthocyans (delphinidin-3-*O*-glucoside, delphinidin-3-*O*-rutinoside, cyanidin-3-*O*-glucoside and cyanidin-3-*O*-rutinoside), catechins, quercetin, myricetin and kaempferol	Gallic acid, *p*-hydroxy-benzoic acid and hydroxycinnamic acid	Proanthocyanidin, ellagitannins and gallotannins	Vitamin A and B_2_	Stilbenoids	Calcium, zinc, magnesium, potassium, gibberellic acids and γ-linolenic acid	[75]
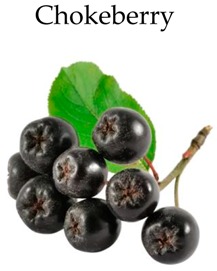	Anthocyans (cyanidin-3-galactoside and cyanidin-3-arabinoside, cyanidin-3-galactoside and cyanidin-3-arabinoside), quercetin glycosides and flavan-3-ols ((−)-epicatechin)	Caffeic acid, hydroxycinnamic acids, chlorogenic acid and neochlorogenic acids	Proanthocyanidins	Vitamin B and C	Stilbenes	Potassium and zinc, β-carotene and β-cryptoxanthin	[33,76,77]
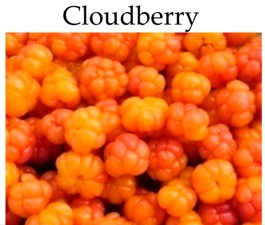	Anthocyanins, flavonols and flavan-3-ols	*p*-Coumaric acid, hydroxycinnamic acids, caffeic acid, ferulic acid and gallic acid	Poanthocyanidins, ellagitannins and ellagic acid	Vitamin C and α-tocopherol	Stilbenes	β-carotene	[78,79,80]
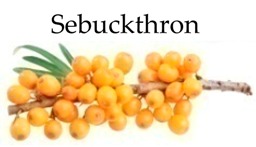	Isorhamnetin (isorhamnetin-rutinoside, isorhamnetin-glycosid) quercetin-rutinoside, quercetin-glycoside and kaempferol	Hydroxyursolic acid		Vitamin A, B_2_, C and E		Carotenoid, calcium, magnesium, potassium and sodium	[81,82]
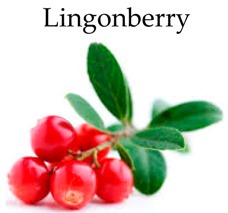	Anthocyanins (cyanidin-3-*O*-sambubioside, delphinidin-3-*O*-galactoside, peonidin-3-*O*-galactoside), quercetin-3-galactoside and flavan-3-ols ((−)-epicatechin)	Ferulic acid, benzoic acid and phenylacetic acid	Proanthocyanidins	Vitamin C and E	Trans-resveratrol		[83,84,85]
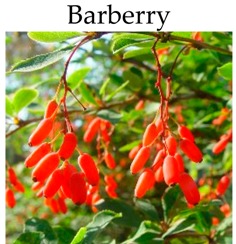	Chrysanthemin, hyperoside, pelargonin and petunidin-3-*O*-beta-d-glucoside	Chlorogenic acid	Tannin	Ascorbic acid and Vitamin K		Isoquinoline alkaloids (berberine, berbamine and palmatine), and β-carotene	[86]
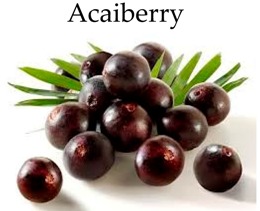	Anthocyanins (cyanidin, delphinidin, malvidin, pelargonidin, and peonidin), isovitexin ,luteolin, quercetin, dihydrokaempferol, chrysoerial and flavan-3-ols	Protocatechuic acid, ferulic acid, syringic acid and vanillic acid			Resveratrol		[87]
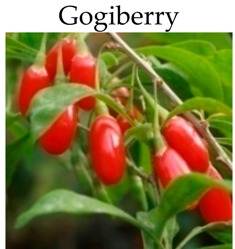	Myricetin, quercetin and kaempferol	*p*-Coumaric acid		Vitamin B1, B2, B3, B6, C and E		*L. barbarum* polysaccharides, amino acids, zinc, iron, copper, calcium, germanium, selenium, phosphorus and β-carotene	[88]
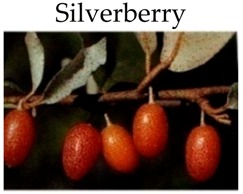	Myricetin and epigallocatechin gallate	Phenolcarboxylic acids, benzoic acid, cinnamic acid and caffeic acid	Condensed tannins	Ascorbic acid		Lycopene, linoleic acid, oleic acid, and stearic acid	[89]
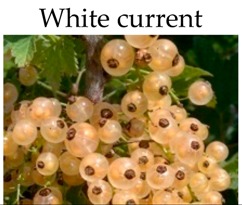	(+)-catechin, and quercetin aglycon	Hydroxybenzoic acid derivative and hydroxycinnamic acid derivatives	Proanthocyanidins				[90]
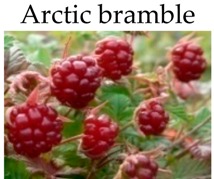	Anthocyanins (cyanidin 3-glucoside, cyanidin 3-rutinoside), (+)-catechin, (−)-epicatechin, quercetin 3-glucuronide and isorhamnetin 3-glucuronide.	Hydroxycinnamic acids	Ellagitannins, ellagic acid and its derivatives				[78]
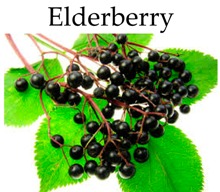	Anthocyanins (cyanidin 3,5-diglucoside and cyanidin 3-glucoside)	Chlorogenic acid		Vitamin C		Lectins	[91]
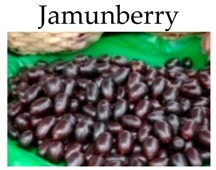	Anthocyanins (3,5-diglucosides of delphinidin, petunidin and malvidin), dihydroquercetin diglucoside, myricetin	Gallic acid and galloyl-glucose ester				Carotenoids and lutein	[92]
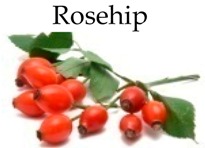	Quercetin, kaempferol and myricetin	Gallic acid, protocatechuic acid, syringic acid, coumaric acid and vanillic acid	Ellagic acid	Vitamin C		Ascorbate, β-carotene, glutathione and α-tocopherol	[93]
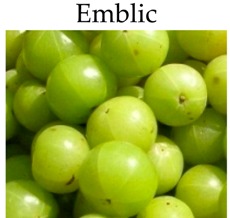	Quercetin	Gallic acid, chebulagic acid, 3-ethyl-gallic acid and geraniin	Ellagic acid, corilagin, and isocorilagin	Vitamin C		Galloyl glucose, amino acids and minerals	[94,95]

A growing amount of evidence indicates that berries contain a wide range of antioxidants (Table 2) [96,97], responsible, at least in part, for their chemopreventive activities. The mechanism of antioxidant activity of berries involvesi) scavenging or quenching of oxygen free radicals; (ii) protection of DNA, proteins, and lipids from reactive oxygen species (ROS); (iii) inhibition of oxidative enzymes [98]; (iv) inhibition of oncogene expression [99,100] and (v) alteration of cellular signaling to regulate the level of antioxidant compounds and enzymes [33].

**Table 2:** Antioxidant capacity, measured as oxygen radical absorbing capacity (ORAC) (µmol Trolox equivalents/g), of various fresh berries.

Berries	Antioxidant Capacity (µmol Trolox Equivalents/g)	References
Chokeberry	158.2	[101,102]
Raspberry	21.4	[103]
Lowbush blueberry	64.4	[103]
Elderberry	145.0	[102]
Blackberry	55.7	[104]
Rabbiteye blueberry	123.4	[104]
Black currant	56.7	[104]
Lingonberry	38.1	[101]
Cranberry	18.5	[101]
Red grape	7.4	[105]
White grape	4.5	[105]
Strawberry	53.03	[106]
Jamunberry	16.4	[92]
Emblic	134.33	[95]

## 3. Bioavailability and Metabolites of Berries

**Figure 2:**
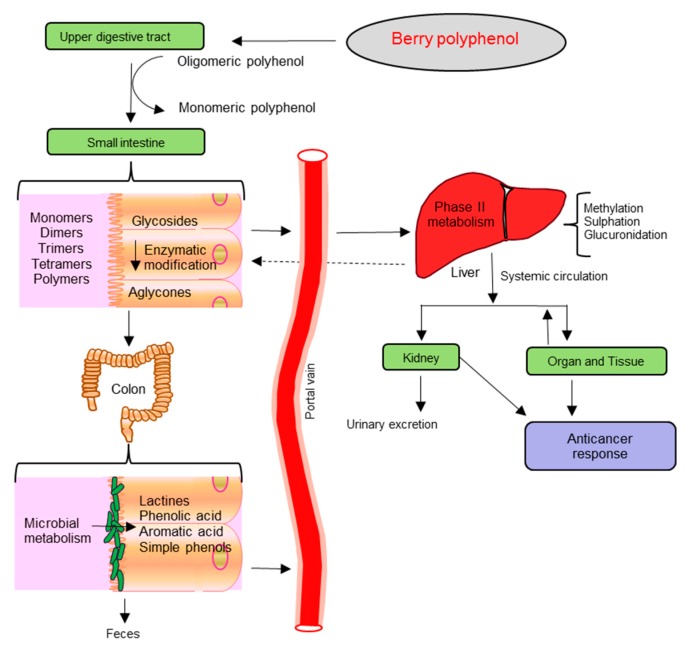
Schematic diagram of organs involved in the absorption and metabolism of berry polyphenols.

It is well established from animal [107,108,109] and human studies [110,111,112] that phenolic compounds of ingested berry phytochemicals survive digestion in the upper digestive tract and reach different parts of the proximal and distal colon in substantial doses (Figure 2). During the absorption process, phenolics are conjugated (usually methylated, sulfated and glucuronidated) in the small intestine and later in the liver, a metabolic detoxification process that facilitates biliary and urinary elimination [113]. Hence, the colonic epithelia can be in contact with both the parent phenolic compounds and their degradation products which are extensively metabolized to simpler phenolics by colonic microbiota [114,115,116]. A few of these metabolites can be detected in urine, feces, blood and tissue, though some phenolic compounds often have very poor bioavailability (Figure 2) [117].

The bioavailability of anthocyanins is very low and only trace levels can be detected in plasma and urine after absorption and excretion. Potential phase I and phase II metabolism appears to be very difficult to evaluate. Anthocyanin metabolites such as cyanidin-3-galactoside, cyanidin-3-glycosides, cyanidin 3-rutinoside, cyanidin glucuronide and glucuronide conjugates, delphinidin 3-glucoside, delphinidin 3-rutinoside, pelargonidin-3-glucoside and malvidin-3-glucoside were detected in urine and serum samples of volunteers who consumed berry extract or juice [111,112,118,119,120].

There are numerous feeding studies with animals and human subjects indicating that polymeric procyanidins are not absorbed [121]. Most of them pass unaltered to the large intestine where they are catabolized by the colonic microflora yielding a diversity of phenolic acids including 3-(3-hydroxyphenyl) propionic acid, 4-*O*-methylgallic acid, *m*-hydroxyphenylacetic acid, *m*-hydroxyphenylvaleric acid and *m*-hydroxybenzoic acid which are absorbed into the circulatory system and excreted in urine [122,123].

Dietary ellagitannins (ET) are hydrolyzed to yield ellagic acid (EA). Later, EA is metabolized by colon bacteria to various urolithins, such as urolithin A (3,8-dihydroxy-6*H*-dibenzo[*b*,*d*]pyran-6-one: UA) and B (3-hydroxy-6*H*-dibenzo[*b*,*d*]pyran-6-one: UB), in the distal part of the small intestine and in the colon [124]. Once absorbed, these microbial metabolites are further subjected to phase II biotransformations in the enterocyte and hepatocyte, producing a combination of urolithin metabolites both in plasma and urine [125].

Furthermore, dietary antioxidants, like vitamin C and E, together with a few carotenoids are absorbed in the upper segments of the intestine [126]. In this perspective, the individual’s gut microbiota will become increasingly relevant in studies on colon cancer with respect to the individual’s bioavailability and ultimately bioactivity of berry polyphenol, metabolizing phenotype or gut metabotype [127,128].

## 4. Biological Activities of Berries against Colon Cancer: *in Vitro* and *in Vivo* Animal Studies

A growing body of evidence has been focused on determining the conceivable mechanisms for colon cancer prevention. Different *in vitro* and *in vivo* models have assessed the efficacy of whole berry extracts, fractionated berry extracts, or purified/commercial berries on different stages of colon cancer (Table 3).

### 4.1. Raspberry

The raspberry (*Rubus* sp., family: Rosaceae), a traditional medical plant, has recently received much attention from both scientists and consumers for its health benefits, due mainly to the high amount of ellagic acid which is a known anticancer agent [129].

Regarding *in vitro* studies, treatment with black raspberry derived anthocyanins suppressed cell proliferation and increased apoptosis in colon cancer HCT-116, Caco-2 and SW480 cells [130]. This effect was partly mediated by mRNA regulation of β-catenin and c-Myc genes [130]. Wang and colleagues found that anthocyanins demethylated tumor suppressor genes such as CDKN2A, SFRP2, SFRP5 and WIF1, through the inhibition of DNA methyl transferase 1 (DNMT1) and DNMT3B in colon cancer cells [130]. A “colon-available” raspberry extract (CARE) was reported to inhibit the key stages of colorectal cancer development [131].

**Table 3:** Anticarcinogenic effects of berry extracts or constituents *in vitro* and/or *in vivo* models of colon cancer.

Berry Extracts/Fraction/Component	Model (Cell Lines or Animal)	Duration and Dose/Intervention	Effects on Colon Cancer	References
**Raspberry**				
***In vitro***				
Polyphenolic-rich extracts	HT-29 and HCT-115 cells	0, 3.125, 6.25, 12.5, 25, 50 μg/mL for 24 h	-Inhibit initiation, promotion and invasion.	[131]
Anthocyanins rich extracts of black raspberry	HCT-116, Caco-2 and SW480 cells	0.5, 5, and 25 μg/mL for 3 days	-Inhibit proliferation. -Suppress DNMT1 and DNMT3B proteins. -Suppress downstream of Wnt pathway. -Induce apoptosis.	[130]
ET and their derivatives from black raspberry seeds	HT-29 cells	5 to 30 μg/mL for 24 and 48 h	-Arrest cell cycle. -Induce apoptosis by extrinsic and intrinsic pathways.	[132]
Aqueous extracts of black raspberry	HT-29 cells	0 to 400 µg/mL for 24 to 48 h	-Inhibit cancer cell growth -Induce apoptosis.	[133]
Red raspberry extracts	LoVo cells	5%, 7.5%, and 10% for 24 to 48 h	-Reduce the survival of cells.	[134]
Black raspberry extracts	HT-29 and HCT-116 cells	25–200 µg/mL for 48 h	-Induce cytotoxic effects.	[59]
Gastrointestinal digestion and colonic fermentation	HT-29 and HT-115 cells	0–50 µg/mL gallic acid equivalents (GAE) for 24 h.	-Exert anti-genotoxic, anti-mutagenic and anti-invasive activity.	[116]
Freeze-dried extracts from black raspberry	HT-29 cells	0.6 and 1.2 mg of extract/mL for 48 h	-Retain their anticancer activity after digestion.	[135]
***In vivo***				
Lyophilized black raspberry	AOM induced Fischer 344 rat	0%, 2.5%, 5%, or 10% (*wt*/*wt*) for 9 to 33 weeks	-Decrease the multiplicity of ACF, total tumors, adenomas, and adenocarcinomas.	[136]
Black raspberry extracts	Interleukin-10 knock-out mouse	5% for 8 weeks	-Decrease colonic ulceration.	[137]
Freeze-dried black raspberry	Apc1638+/− mice and Muc2−/− mice	10% for 12 weeks	-Lower tumor incidence and multiplicity.	[138]
	DSS induced male C57BL/6J mice	5%–10% for 7–14 days	-Ameliorates ulcerative colitis. -Suppresses inflammation.	[139]
	DSS induced male C57BL/6J mice	5% for 28 days	-Suppresses colonic ulceration by correcting promoter hypermethylation of suppressor genes.	[140]
**Blueberry**				
***In vitro***				
Dried extracts and fractions	HT-29 and Caco-2 cells	50–10,000 µg/mL for 48 h	-Inhibit cancer cell proliferation. -Induce apoptosis.	[141]
Ethanol/water extracts	HT-29 cells	0.025%–0.5% dry wt for 24 h	-Exert antiproliferative activity.	[142]
Anthocyanin-rich extracts	Caco-2 cells	0.1–100 nM for 1 h	-Act as an intracellular antioxidant.	[143]
	DLD-1 and COLO205 cells	50–250 μg/mL for 24 h	-Repress the proliferation. -Induce apoptosis.	[144]
Blueberry extracts	HT-29 and HCT116 cells	25–200 µg/mL for 24 to 48 h	-Inhibit cancer cell proliferation.	[145]
IVD and colonic fermentation	HT-29 or CRL-1790 cells	10, 25, 50, 75 or 100 µg/mL for 24 to 48 h	-Alter antiproliferative and antioxidant activity after digestion.	[146]
Delphinidin	HCT116 cells	30–240 mM for 48 h	-Inhibit cancer cell growth. -Induce apoptosis. -Arrest cell cycle.	[147]
Anthocyanin-enriched fractions	HT-29 cells	50–150 µg/mL for 6 h	-Induce apoptosis.	[148]
Pterostilbene	HT-29 cells	50 µM for 4 h	-Suppresses cell growth. -Suppresses inflammation.	[149]
***In vivo***				
Pterostilbene	AOM induce Fisher 344 male	40 p.p.m. (0.004%) for 45 weeks	-Reduce tumor multiplicity, by inhibiting the Wnt/β-catenin signaling pathway.	[149]
Blueberry extracts	AOM induce Fisher 344 male	50 g/kg for 13 weeks	-Reduce formation of AOM-induced ACF and increase in hepatic GST activity.	[150]
Blueberry husks and mixture of three probiotic	DSS treatment rat	50 g /kg diet for 6 months	-Reduce colonic ulcers and dysplastic lesions.	[151]
**Grape**				
***In vitro***				
Grape seed proanthocyanidin extract	Caco-2 cells	10–100 µg/mL for 24 h	-Inhibits cancer cell proliferation. -Reduces PI3k/PKB signaling pathway. -Induces caspase-3 dependent apoptosis.	[152]
Anthocyanin-rich extracts	HT-29 cells	0 to 200 μg/mL for 48 h	-Inhibit cell proliferation.	[153]
	HT-29 cells	10–75 µg of monomeric anthocyanin/mL for 24–72 h	-Induce anti-proliferative activity.	[154]
	HT-29 cells	500 µg/ml for 72 h	-Protect DNA damaging properties of topoisomerase poisons.	[155]
Obacunone and obacunone glucoside (OG) from seeds of marsh white grape	SW480 cells	6.25, 12.5, 50, and 100 µM for 24, 48 and 72 h	-Induce intrinsic pathway of apoptosis.	[156]
Grape waste	Caco-2 cells	0.5, 1.5, 10, 50, or 100 mL/L for 24 h	-Induce strong antiradical and antiproliferative activity. -Arrest cells cycle.	[157]
***In vivo***				
Anthocyanin-rich extracts	AOM treated Fischer 344 male rats	3.85 g of monomeric anthocyanin/kg body weight for 14 weeks	-Inhibit colonic aberrant crypt foci formation.	[158]
Total pholyphenolic extracts	DMH induced F344 rats	0.11 % (*w*/*w*) for 16 weeks	-Decrease number of adenomas.	[159]
Proanthocyanidin-rich dietary fiber	C57BL/6J mice	10 mL/kg body weight for 2 weeks	-Alters the expression of tumor suppressor genes and proto-oncogenes. -Modulates genes associated with lipid biosynthesis, energy metabolism, cell cycle, and apoptosis.	[160]
**Strawberry**				
***In vitro***				
Crude extracts and purified compounds	HT-29 and HCT-116 cells	250 μg/mL (crude extract) and 100 μg/mL (pure compounds) for 48 h	-Inhibit cell proliferation.	[161]
Polyphenol-rich extracts	Caco-2 cells	25, 50, and 75 μg of GAE /mL	-Show anti neoplastic activity.	[162]
Strawberry extracts	HT-29 cells	0.025%, 0.05%, 0.25%, 0.5% for 24 h	-Organically grow strawberry extracts show higher antiproliferative activity.	[163]
	HT-29 and HCT-116 cells	25–200 µg/mL for 24 to 72 h	-Inhibit cancer cell proliferation.	[145]
*IVD* and fecal fermentation	HT-29 and HT-115 cells	0–50 µg/mL GAE for 24 h	-Exerts anti-genotoxic, anti-mutagenic and anti-invasive activity.	[116]
Extracts from strawberries treated with essential oils	HT-29 cells	3 mg/mL for 24 h to 96 h	-Exhibit strong radical scavenging capacity and antiproliferative activity.	[164]
Kaempferol	HT-29 cells	0 or 60 μmol/L for 24 to 72 h	-Inhibit cancer cells growth. -Arrest cell cycle.	[165]
ET extracts, EA and UA.	Human 293T cells	10–1000 µg/mL for 48 h	-Inhibit the canonical Wnt signaling pathway.	[166]
***In vivo***				
Freeze-dried strawberry	AOM/DSS induced male Crj: CD-1 mice	2.5%, 5.0% or 10.0% for 20 weeks	-Reduce proinflammatory mediators and oncogenic signaling pathways.	[167]
**Bilberry**				
***In vitro***				
Ethanol extracts	HCT-116 cells	4 mg/mL for 24 or 48 h	-Inhibit cancer cell proliferation.	[168]
Anthocyanin-rich extracts	HT-29 cell	25–75 μg/mL (equivalents as cyanidin 3 glucoside) for 48 h	-Inhibit cell proliferation	[153]
	HT-29 cells	0–60 mg/mL for 24 h	-Inhibit cancer cell proliferation. -Induce apoptosis.	[168]
	HT-29 cells	50–500 µg/mL for 72 h	-Suppress the DNA-damaging properties.	[155]
	HT-29 cells	5–500 µg/mL for 1 to 24 h	-Exhibit cytotoxicity. -Decrease DNA damage and ROS level.	[169]
Anthocyanin-rich extracts	HT-29 and NCM460 cells	10–75 µg of monomeric anthocyanin/mL for 24–72 h	-Inhibit cancer cell proliferation.	[154]
Anthocyanin-rich extracts	Caco-2 cells	0.1–100 nM for 1 h	-Exert potent intracellular antioxidant activity.	[143]
***In vivo***				
Anthocyanin-rich extracts	AOM treated Fischer 344 male rats	3.85 g of monomeric anthocyanin/kg for 14 weeks	-Decrease the number of total and large ACF.	[158]
Mirtoselect and cyanidin-3-glucoside	Apc Min/+ mouse	0.03%–0.3% for 12 weeks	-Decrease the total numbers of intestine adenomas.	[170]
Freeze-dried bilberry	Apc Min/+ mouse	1564 mg/kg for 10 weeks	-Inhibit the formation of intestinal adenoma.	[171]
**Cranberry**				
***In vitro***				
Cranberry presscake and whole cranberry extract	HT-29 cells	0–600 mg/mL for 4 days	-Exhibit antiproliferative activity.	[172]
Cranberry extracts and polyphenol fraction	HCT-116, SW480 and SW620 cells	50–200 μg/mL (extract) and 6.5–78.8 μg/mL (fractions) for 48 h	-Enhance antiproliferative activity.	[173]
***In vivo***				
Cranberry extracts and dried cranberry	DSS induced murine colitis	0.1% creanberry extract and 1.5% dry cranberry for 1 week	-Prevent colitis. -Decrease inflammatory cytokines.	[174]
Cranberry products	AOM induced Fisher 344 male	50 g/kg for 17 weeks	-Inhibit colonic ACF formation.	[150]
Fr6 and purified proanthocyanidin	Xenografts Balb/c mice	100 mg/kg proanthocyanidin and 250 mg/kg Fr6 for every 2 days for 3 weeks	-Decrease tumor growth and volume.	[175]
Juice of high-bush cranberry	DMH treated mouse	65% gilaburu pulp and 45% water (pH: 3.09) for 30 weeks	-Inhibit tumor lesion at the initiation stage.	[176]
**Mangosteen**				
***In vitro***				
α-Mangostin and other xanthones extracts	HCT-116 cells	2.5–30 μg/mL for 48 h	-Induce cytotoxicity and apoptosis.	[71]
α-Mangostin	HCT-116 cells	14.8–25.6 µM for 24 h	-Inhibit proliferation. -Induce apoptosis and arrest cell cycle.	[177]
	DLD-1 cells	0 to 20 µM for 24 h	-Inhibit proliferation. -Induce apoptosis.	[178]
	HT-29 cells	6–12 μM for 24 h	-Exert anti-proliferative activity. -Decrease Bcl-2 and β-catenin expresion.	[179]
γ-Mangostin	HT-29 cells	10–200 μM for 24 h	-Inhibits cancer cell proliferation. -Induces apoptosis and increases ROS.	[180]
***In vivo***				
Extracts of mangosteen pericarp	Established subcutaneous tumor of HCT-116 cells in NCR nude mice	0.25% and 0.5% for 20 days	-Inhibit tumor growth and fewer blood vessels in tumor.	[181]
α-Mangostin	HT-29 colon cell xenogrft Balb/c nu/nu mice	900 mg /kg for 2 or 4 weeks	-Decrease tumor masses and anti-apoptotic protein, Bcl-2, and β-catenin.	[179]
	Her2/CT26 colon cell xenografts mice	20 mg/kg daily for 3 days	-Reduce tumor growth by autophagy activation.	[182]
	DMH induce Fisher 344 rats	0.02% and 0.05% for 5 weeks	-Inhibit development of ACF. -Decreases dysplastic foci, β-catenin accumulated crypts and lower PCNA.	[183]
Crude methanolic extract	Mice were implanted with NL-17 cells	0–250 mg/kg for 14 days	-Increase life span by decreasing tumor growth.	[184]
**Blackberry**				
***In vitro***				
Blackberry extract	HT-29 and HCT-116 cells	25–200 µg/mL for 24 to 48 h	-Exert antiproliferative effects.	[145]
Anthocyanin-rich extracts from hull and crude blackberry	HT-29 cells	13.6 to 49.2 µg of monomeric anthocyanins/mL for 48 to 72 h	-Induce significant antioxidant and antiproliferative activity.	[185]
Anthocyanin-rich extracts from crude blackberry	Caco-2 cells	0.8, 1.6, 3.1, 6.3, 12.5 and 25 µg/mL for 24 h	-Inhibit peroxyl radical induced apoptosis.	[186]
***In vivo***				
Blackberry products	AOM induced Fisher 344 male	50 g/kg for 17 weeks	-Inhibit colonic ACF formation.	[150]
**Blackcurrant**				
***In vitro***				
Black currant press residue extracts	Caco-2, HT-29, and HCT-116 cells	0–125 μg GAE/mL for 24 to 48 h	-Suppress cancer cell proliferation.	[187]
Black currant extracts	HT-29 cells	0.025% to 0.5% dry wt for 24 h	-Exert antiproliferative effect.	[142]
Methanol extracts of blackcurren	HT-29 cells	0–60 mg/mL for 24 h	-Diminish cell proliferation via the p21WAF1 pathway.	[168]
*IVD* digestion and fecal fermentation	HT-29 and HT-115 cells	0–50 µg/mL GAE for 24 h	-Exert anti-genotoxic, anti-mutagenic and anti-invasive activity.	[116]
**Chokeberry**				
***In vitro***				
*In vitro* gastric and pancreatic digestion of chokeberry juice	Caco-2 cells	0 to 800 µM for 2 h a day for 4 days period	-Inhibit cell proliferation. -Arrest cell cycle at G2/M phase -Upregulate tumor suppressor CEACAM1 gene expresion.	[125]
Anthocyanin-rich extracts	HT-29 cells	0 to 200 μg/mL for 48 h	-Suppress cancer cell proliferation.	[153]
	HT-29 cells	10–75 µg of monomeric anthocyanin/mL for 24–72 h	-Inhibit cancer cell proliferation.	[154]
	HT-29 cells	50 μg monomeric anthocyanin/mL for 24 h	-Inhibit cell proliferation. -Block the cell cycle and increase cell cycle regulatory protein.	[188]
***In vivo***				
Anthocyanin-rich extracts	AOM treated Fischer 344 male rats	3.85 g of monomeric anthocyanin/kg for 14 weeks	-Inhibit colonic ACF formation.	[158]
**Cloudberry**				
***In vitro***				
Polyphenol-rich extracts	Caco-2 cells	25, 50, and 75 μg of GAE/mL	-Inhibit cancer cell proliferation.	[162]
Methanolic extraction	HT-29 cells	0–60 mg/mL for 24 to 48 h	-Disrupt cell proliferation. -Increases p21WAF1pathway.	[168]
***In vivo***				
Freeze dried cloubberry	Apc Min/+ mouse	1564 mg/kg for 10 weeks	-Inhibits the formation of intestinal adenoma.	[171]
**Seabuckthorn**				
***In vitro***				
Polyphenol rich extracts	HT-29 cells	0.025%–0.5% dry wt for 24 h	-Inhibit cancer cell proliferation.	[142]
Isorhamnetin	HT-29, HCT-116 and SW480 cells	0–80 μmol/L for 3 days	-Decreases cancer cell proliferation. -Inhibits signaling pathway and arrests cell cycle.	[189]
***In vivo***				
Seabuckthorn seed oil	PhIP exposure Wistar rats	2 to 8 mL/kg body wt for 12 to 36 h	-Improves oxidative stress and decreases abnormal cancer related gene expression.	[190]
**Lingonberry**				
***In vitro***				
Polyphenol-rich extracts	Caco-2 cells	25, 50, and 75 μg of GAE/mL	-Induce antiproliferative activity.	[162]
Anthocyanin-rich extract	HT-29 cells	0.025%–0.5% dry wt for 24 h	-Suppress the growth of cancer cells.	[142]
	HT-29 cells	0–60 mg/mL for 24 to 48 h	-Decrease cell proliferation proliferation via p21WAF1pathway.	[168]
***In vivo***				
Freeze dried lingonberry	Apc Min/+ mouse	1564 mg/kg for 10 weeks	-Decrease adenoma formation.	[171]
**Barberry**				
***In vitro***				
Berberine	SW480 cells	5–50 µM for 12–72 h	-Suppresses cells growth. -Arrests cell cycle. -Induces apoptosis. -Inhibits angiogenesis and inflammation markers.	[191]
**Acai Berry**				
***In vitro***				
Polyphenolic extracts	SW480, HT-29 and CCD-18Co cells	5–20 mg/L for 48 h	-Suppress cells growth. -Show anti-inflammatory activity.	[192]
***In vivo***				
Spray-dried acai powder	DMH in male Wistar rats	2.5% or 5.0% acai power for 20 weeks	-Reduces the number of aberrant crypts, invasive tumors and tumor multiplicity.	[193]
**Goji berry**				
***In vitro***				
*Lycium barbarum* polysaccharides	SW480 and Caco-2 cells	100–1000 mg/L for 1, 2, 3, 4,or 5 days	-Decreases cells growth by intrupting cell cycle.	[194]
**Silverberry**				
***In vitro***				
Extracts from seed and flesh of cherry silverberry	HT-29 cells	Seed extract (100–1600 g/mL) and flesh extract (200–3200 g/mL) for 48 h	-Exert anti-inflammation and anti-proliferation activities.	[195]
**White currant**				
***In vivo***				
Freeze dried white currant	Min mice	10% for 10 weeks	-Prevents cancer initiation and progression.	[196]
**Arctic bramble**				
***In vitro***				
Polyphenol-rich extracts	Caco 2 cells	25, 50, and 75 μg of GAE/mL	-Reduce cancer cell proliferation.	[162]
**Elderberry**				
***In vitro***				
Anthocyanin-rich extracts	HT-29 cells	0 to 200 μg/mL for 48 h	-Inhibit cancer cell proliferation.	[153]
**Jamun berry**				
***In vitro***				
ETs rich jamun berry extracts	Human 293T cells	10–1000 µg/mL for 48 h	-Exert chemopreventive activity. -Inhibit the canonical Wntsignaling pathway.	[166]
**Rosehip**				
***In vitro***				
Polyphenol rich extracts	HT-29 cells	0.025,0.05, 0.25, and 0.5% dry wt for 24 h	-Inhibit cancer cell proliferation.	[142]
		62.5–1000 g/L for 24 h	-Suppress cancer cell growth.	[93]
**Emblic**				
***In vitro***				
Ethanolic extracts	HT-29 cells	10-100 μg/mL for 48 h	-Inhibit cancer cell proliferation.	[197]
Water extract	COLO320 cells	0, 20, 40, 80, or 160 μg/mL PE for 24, 48, 72, or 96 h	-Suppress necrosis and delays mitotic progression. -Induce apoptosis.	[198]

In fact, CARE was able to inhibit both initiation through protecting hydrogen peroxide induced DNA damage, and promotion through decreasing cell population in the G1 phase inHT-29 cells; moreover, in HT-115 colon cancer cells, it reduced the number of cells entering the cell cycle and inhibited cell invasion [131]. Aqueous extract of Korean black raspberry was reported to constrain HT-29 colon cancer cell growth by inducing apoptosis and inhibiting DNA synthesis [133]. This extract induced cleavage of poly(ADP-ribose) polymerase (PARP) and increased the activity of caspase-3, -7, and -9, suggesting that the induction of apoptosis was mediated by the activation of the caspase pathway [133]. Similarly, in colon cancerHT-29 and HCT-116 cells, black raspberry extracts induced cytotoxic effects, exerting significant pro-apoptotic effects of the cyclooxygenase-2 (COX-2) expressing HT-29 cells [145]. Recently, Cho *et al.*, investigated the chemopreventive activity of ET and their derivatives from black raspberry seeds on HT-29 cells [132]. They found that ET, hydrolyzed to EA and further metabolized to UA and UB, showed anti-cancer activity by: (i) inhibiting cell proliferation; (ii) arresting the cell cycle at G1 and G2/M phase and (iii) inducing apoptosis by both extrinsic and intrinsic apoptotic pathways [132]. Finally, it should be take into account that the antiproliferative activity of black raspberry extracts on HT-29 cells is significantly influenced by cultivar, production site, stage of maturity and the source material [135]. God *et al.*, reported that red raspberries exhibited cytotoxic activity in LoVo cells, where antioxidants play a minor role and no apoptotic effect was observed [134].

As far as *in vivo* studies are concerned, lyophilized black raspberry consumption has been reported to decrease the multiplicity on azoxymethane (AOM) induced dysplastic aberrant crypt foci (ACF), total tumors, tumor volumes, adenomas and adenocarcinomas in Fischer 344 rats [136]. In addition, black raspberries significantly reduced urinary 8-hydroxy-2’-deoxyguanosine (8-OHdG) levels and altered oxidative stress markers, and markers of DNA damage [136]. Bi *et al.*, investigated the chemopreventive effects of freeze dried black raspberries in two mouse models of human colorectal cancer, namely *Apc*1638+/− and *Muc*2−/− [138]. They found that 12-week feeding of black raspberries significantly inhibited intestinal tumor formation by reducing tumor incidence and tumor multiplicity in both models [138]. Mechanistic studies informed that black raspberries inhibited tumor development by (i) suppressing β-catenin signaling in *Apc*1638+/− mice; (ii) reducing chronic inflammation in *Muc*2−/− mice and (iii) inhibiting intestinal cell proliferation in both models [138]. Furthermore, black raspberries inhibited colonic ulceration associated with colon cancer in interleukin-10 (IL-10) knock-out mouse by suppressing the nuclear translocation of β-catenin [137]. Dietary supplementation of freeze dried black raspberries markedly reduced dextran sodium sulfate (DSS) induced acute injury to the colonic epithelium and colonic ulceration in C57BL/6J mice [140]. Wang and colleagues found that black raspberries suppressed colonic ulceration by (i) decreasing NF-kB p65 protein expression; (ii) reducing the level of DNMT3B; (iii) attenuating promoter methylation of tumor suppressor genes in the Wnt pathway; and (iv) decreasing translocation of β-catenin to the nucleus [140]. Moreover, black raspberries induced anti-inflammatory activity by suppressing tissue levels of COX-2 as well as proinflammatory cytokine tumor necrosis factor-alpha (TNF-α), prostaglandin E2 (PGE2) and IL-1β in DSS-induced ulcerative colitis in male C57BL/6J mice [139].

### 4.2. Blueberry

The blueberry (*Vaccinium corymbosum* L., family: Aricaceae) is rich in polyphenols such as anthocyanins, flavonols, tannins and phenolic acids which show the potentiality to prevent cancer through their biological activities [141]. In the last decade, the blueberry has become more famous for its nutritional value and human benefits.

Blueberry extract was reported to inhibit human colon cancer HT-29 and HCT-116 cell proliferation at high concentrations [145] and human colon cancer Caco-2 cells growth at low concentrations [162]. It was shown that the inhibition of cancer cell proliferation was highly correlated with the levels of polyphenols, flavonoids and antioxidant activities [142,145]. For example, anthocyanin rich extracts of the blueberry act as potent intracellular antioxidants in Caco-2 cells, even at very low concentrations [143]. Similarly, delphinidin and malvidin, isolated from a blueberry anthocyanin-rich extract, repressed the proliferation of DLD-1 and COLO205 human colorectal cancer cells via induction of apoptosis [144]. Yi *et al.*, investigated the proliferative effect of dried extracts and different fractions of blueberries in HT-29 and Caco-2 cells. They found that anthocyanin fractions presented significant antiproliferative activity and increased DNA fragmentation, indicating the induction of apoptosis [141]. *In vitro* digestion (IVD) and colonic fermentation of blueberry polyphenols were tested using normal human colonic epithelial CRL 1790 cells and human colorectal cancer HT-29 cells [146]. A high stability of total polyphenols and anthocyanins during the simulated gastric digestion step was found but intestinal digestion decreased polyphenol and anthocyanin contents compared to the non-digested samples [146]. The catabolic products showed lower antiproliferative and antioxidant effects in HT-29 or CRL-1790 cells [146] and suggested that colonic fermentation may alter the biological activity of blueberries. In another study, anthocyanin-enriched fractions from blueberry-induced apoptosis in HT-29 cells by increasing DNA fragmentation and caspase-3 activity [148]. This study also noticed that anthocyanin-enriched fractions decreased quinine reductase and glutathione S-transferase (GST) activities compared with untreated cells [148]. Moreover, delphinidin inhibited HCT-116 cells growth by: (i) inducing apoptosis by cleavage of PARP, activation of caspase-3, -8, and -9 and alteration of B-cell lymphoma 2 associated X (Bax)/B-cell lymphoma 2(Bcl-2) ratio; and (ii) arresting cell cycle at G2/M phase [147]. Delphinidin-induced apoptosis and cell cycle arrest were associated with suppression of NF-κB pathway [147]. Pterostilbene, a primary antioxidant component of blueberries, suppressed HT-29 cell proliferation as well as inflammation by down regulating the levels of β-catenin, cyclin D1, c-MYC and phosphorylation of p65 [149].

In an *in vivo* study, blueberries and pterostilbenes reduced the incidence and multiplicity of ACF formation with AOM induced rats [149,150] and significantly increased hepatic GST activity [150]. Paul and coworkers noticed that pterostilbenes inhibited colon tumorigenesis by regulating the Wnt/β-catenin-signaling pathway and the inflammatory responses. They found that pterostilbenes (i) decreased cell proliferation markers, such as proliferating cell nuclear antigen (PCNA); (ii) down-regulated the expression of β-catenin and cyclin D1; (iii) reduced the expression of inflammatory enzymes, inducible nitric oxide synthase (iNOS) and COX-2, and inflammatory cytokines, TNF-α and IL-1β and (iv) decreased nuclear staining of phospho-p65 [149]. Artificial colorectal tumors were created in rats by cyclic treatment with DSS. Blueberry husks and a mixture of three probiotic strains (*Bifidobacterium infantis* DSM 15159, *Lactobacillus gasseri* DSM 16737 and *Lactobacillus plantarum* DSM 15313) were used for treatment. This mixture reduced the number of dysplastic lesions and mucosal ulcers, lowered the proportion of butyric acid and decreased the haptoglobin levels in rat colon [151].

### 4.3. Grape

Grapes (*Vitis vinifera* L.) are one of the most popular and consumed berries worldwide belonging to the Vitaceae family. They are rich in phytochemicals, mostly phenolic acids, stilbenes (resveratrol), anthocyanins, and proanthocyanidins.

Grape seed proanthocyanidin extract (GSPE) has been reported to significantly hinder cell viability and increase apoptosis in Caco-2 cells, but not in normal colon cells [152]. The increased apoptosis in GSPE-treated Caco-2 cells was correlated with an attenuation of PI3-kinase (p110 and p85 subunits) and PKB Ser (473) phosphorylation [152]. Likewise, anthocyanin-rich grape extracts inhibited colon cancer derived HT-29 cells growth [153,154] and suppressed doxorubicin-mediated enhancement of levels of topoisomerase II covalently linked to DNA in HT-29 cells [155]. Furthermore, obacunone and obacunone glucoside isolated from seeds of marsh white grapes generated cytotoxicity in human colon cancer SW480 cells by inducing apoptosis through activation of cytochrome-c mediated intrinsic apoptosis pathway [156]. The increase of caspase-3 and -9 activities and the reduction of Bcl-2/Bax gene transcription ratio were also confirmed in the involvement of apoptosis. In addition, obacunone and obacunone glucoside arrested cells at G1 and G2/M phase by activation of p21 protein [156]. Nevertheless, grape waste, that contains a high amount of polyphenols, showed a strong antiradical and antiproliferative activity in Caco-2 cells, and a significant reduction of cells in G1 phase [157].

Regarding *in vivo* studies, anthocyanin-rich extracts from grapes reduced colonic ACF formation induced by AOM in male Fischer 344 rats compared with the control group [158]. The extracts down-regulated COX-2 gene expression in colonic mucosa but there was no change in cellular proliferation [151]. Total polyphenolic extracts from red wine were also reported to reduce the number of adenomas on 1,2-dimethylhydrazine (DMH) induced colon carcinogenesis in rats [159]. In another study, lyophilized red grape pomace, containing proanthocyanidin-rich dietary fiber, decreased the risk of CRC by persuading genetic and metabolic variation in female C57BL/6J mice. Grape antioxidant dietary fibers induced (i) up-regulation of tumor suppressor genes, NBL1, and of apoptosis genes, BFAR and CARD14; (ii) down-regulation of tumor development genes (such as TNFAIP8L1 and TNF), proto-oncogenes (such as RASSF4, RAP2C, and RAP2B), and cell cycle genes, (iii) modulation of some genes, including lipid biosynthesis ELOVL5, energy metabolism G6PC2, PDK4, SUCLG2, and SUCNR1 [160].

### 4.4. Strawberry

Strawberries (*Fragaria* X *ananassa* Duch.; family: Rosaceae) are known as a functional food with a remarkable combination of phytochemicals (ellagic acid, anthocyanins, quercetin, and catechin), vitamins (ascorbic acid and folic acid), mineral and fibers [66,67,199].

Strawberry fruit crude extracts and purified compounds have been reported to inhibit the growth of human colon cancer HT-29 and HCT-116 cells [161]. Polyphenol-rich extracts also exhibited greater antiproliferative activity in Caco-2 cells [162]. Olsson *et al.*, investigated the inhibitory effect of five cultivars of strawberries in colon cancer HT-29 cells [163]. This study noticed that organically grown strawberries had a higher antiproliferative activity at the highest concentration compared with the conventionally grown type. The presence of a higher content of secondary metabolites in organically grown strawberries was responsible for anticarcinogenic properties [163]. In another study, strawberry extracts increased the inhibition of HT-29 and HCT-116 cells proliferation and stimulated apoptosis of the COX-2 expressing HT-29 cells [145]. Moreover, strawberries retained their biological activity after *IVD* and fermentation, and the digestive products showed significant anti-genotoxic, anti-mutagenic and anti-invasive activity on HT-29 and HT-115 cells [116]. Furthermore, breakdown products of strawberry extract including tyrosol and 4’-hydroxyphenylacetic acid were reported to modulate cellular processes associated with colon cancer [116], while strawberries treated with essential oils, namely thymol, menthol, or eugenol, exhibited strong radical scavenging capacity and antiproliferative activity in HT-29 cells compared with untreated fruits [164]. Kaempferol is a flavonoid found in the strawberry. The anti-carcinogenic effects of kaempferol have been described in HT-29 cells due to the induction of cell cycle arrest at G1 and G2/M phase as well as suppression of the activity of CDK2, CDK4, cyclin D1, cyclin E, cyclin, A Cdc25C, Cdc2, cyclin B1, retinoblastoma protein (Rb) and Wnt signaling pathway [165,166].

Only few *in vivo* studies have investigated the effects of strawberry consumption on CRC. Dietary lyophilized strawberries were reported to prevent inflammation-induced colorectal carcinogenesis in Crj: CD-1 mice. Strawberries also reduced TNF-α, IL-1β, IL-6, COX-2, iNOS and PGE2 expression and decreased phosphorylation of PI3K, Akt, ERK and NF-κB pathway, suggesting that strawberries targeted proinflammatory mediators and oncogenic signaling for carcinogenesis suppression [167].

### 4.5. Bilberry

Bilberry fruit (*Vaccinium myrtillus* L.) belongs to the Ericaceae family and has been used in folk medicine for centuries. It has been extensively studied as a source of anthocyanins and phenolic composition as well as for its antioxidant activity [69,96,200].

Bilberry anthocyanins have been reported to act as a powerful intracellular antioxidant in Caco-2 cells only at low concentrations [143]. In human colon cancer HT-29 cells, they inhibited the growth [153,154,168] and suppressed the cellular viability via amelioration of oxidative DNA damage, suppression of ROS level and elevation of GSH content [169]. However, there was little effect of these extracts on non-transformed colon epithelial NCM460 cells [154]. Bilberry phenolic extracts were reported to induce antiproliferative effect in HT-29 cells by increasing the expression of the p21WAF1 pathway [168]. Esselen and coresearchers found that bilberry extracts significantly suppressed the DNA strand breaking effects of camptothecin and doxorubicin (topoisomerase poisons applied during chemotherapy) in HT-29 cells [155]. Bilberry extracts were also found to be the most effective among ethanol extracts of 10 edible berries in overwhelming the growth of human colon cancer HCT-116 cells [201].

In an *in vivo* model, anthocyanin rich extracts of bilberries reduced the incidence of ACF in rat colon induced by AOM. This effect was mediated, at least in part, by decreasing colonic cellular proliferation, lowering COX-2 mRNA expression, and reducing the fecal bile acids and urinary 8-OHdG level [158]. In addition, dietary administration of freeze dried bilberry fruit significantly reduced the total number of intestinal adenomas in Min (multiple intestinal neoplasia)/+ mice [171]. Cooke *et al.*, investigated the chemopreventive effect of cyanidin-3-glucoside and Mirtoselect, an anthocyanin mixture from bilberries, against intestinal adenoma development in the Apc Min mouse model (a genetic model of human FAP). Ingestion of either cyanidin-3-glucoside or Mirtoselect reduced adenoma load dose-dependently compared to controls groups. They also noticed that anthocyanins were identified at the analytical detection limit in plasma, intestinal mucosa and urine [170].

### 4.6. Cranberry

The cranberry (*Vaccinium macrocarpon* Aiton, family: Ericaceae), a traditional folk remedy, has gained importance over the past decade due to its phytochemicals, particularly flavonol glycosides, anthocyanins, proanthocyanidins (condensed tannins), and organic and phenolic acids [173,202].

The antiproliferative activity of cranberry extracts and various fractions were observed against a series of colon cancer cell lines including HCT-116, SW480 and SW620. The total polyphenol fraction was the most active fraction against all cell lines compared to other cranberry extracts [173]. A flavonoid-rich fraction 6 (Fr6) and a more purified proanthocyanidin rich fraction were isolated from cranberry presscake and whole cranberries. Fr6 and proanthocyanidin significantly inhibited the proliferation of colon cancer HT-29 cells [172].

Cranberry products exhibited chemopreventive effects, also *in vivo*, against AOM induced colon carcinogenesis in male Fisher 344 rats. Dietary feeding of cranberry juice instead of drinking water contributed to significant reductions in the formation of ACF. Moreover, hepatic GST activities were significantly higher compared to control animals [150]. Recently, Xiao *et al.*, reported that cranberry extracts and dried cranberries prevented experimental colitis induced by DSS in mice by reducing shortening of colon length, colonic myeloperoxidase activity and decreasing production of pro-inflammatory cytokines, TNF-α and IL-1β [174]. In another study, Fr6 and purified proanthocyanidin rich fraction decreased tumor growth and volume in HT-29 cell line xenograft mice [175]. In addition, juice of high-bush cranberries prevented the progression of DMH induced colonic lesion numbers in the mouse colon at the initiation stage of colon cancer [176].

### 4.7. Mangosteen

The mangosteen (*Garcinia mangostana* L., family: Clusiaceae) is known as a medicinal plant due to its remarkable pharmacological effects. It contains high amounts of α-, β-, and γ-mangostin xanthones, its major bioactive compounds [71,72,73].

α-Mangostin and xanthone extracts have shown potent cytotoxicity in human colon cancer HCT-116 cells by inducing the mitochondrial pathway of apoptosis [181]. These extracts also inhibited 3 key steps in tumor metastasis including cell migration, cell invasion and clonogenicity. In addition, they up-regulated the MAPK/ERK, c-Myc/Max, and p53 cell signalling pathways [181]. The inhibitory effects of α-mangostin and its related five compounds (3-isomangostin, xanthone, 9,10-anthraquinone, 9-anthracenecarboxylic acid and anthracene) were investigated in human colon cancer HCT-116 cells. Among the tested compounds, α-mangostin was the most potent inhibitor, suppressing cell growth, inhibiting the activity of cellular DNA topoisomerases, interrupting cell cycle in the G2/M phase and inducing apoptosis [177]. In another study, α- mangostin inhibited HT-29 cell proliferation and decreased Bcl-2 and β-catenin expression [179]. Nakagawa *et al.*, investigated in colon cancer DLD-1 cells the cytotoxic effects of α-mangostin, which were mediated via induction of caspase independent apoptotic pathway. They also found that α-mangostin induced apoptosis was mediated via mitochondria pathway with the release of endonuclease-G and increases of miR-143 expression [178]. Furthermore, α-mangostin acted synergistically with low dose 5-FU, increasing DLD-1 growth inhibition [180]. Finally, γ-mangostin demonstrated anticancer activity in HT-29 cells by producing intracellular ROS and inducing apoptosis [180].

Dietary administration of an extract from mangosteen pericarp containing α- and γ-mangostin caused significant growth inhibition of the subcutaneous tumor of colorectal HCT-116 xenografts in mice [181]. In addition, α-mangostin reduced tumor mass and the concentrations of Bcl-2and β-catenin of colon cancer HT-29 xenograft mice compared to the control group. In this study, xanthones and their metabolites were identified in mice serum, tumor, liver and feces [179]. Oral administration of α-mangostin also decreased the growth of colon cancer Her2/CT26 xenografts in mice. The anti-tumor effect of α-mangostin was attributed to autophagic activation rather than induction of endoplasmic reticulum stress [182]. The crude α-mangostin significantly inhibited the induction and development of ACF formation in DMH induced in Fisher 344 rats [183], where fewer dysplastic foci, decreased PCNA in colon and β-catenin accumulated crypts were also detected [183]. Furthermore, crude methanolic extract from mangosteen pericarp suppressed tumor growth and significantly increased the life span by nearly double in BALB/c mice bearing colon cancer NL-17 xenografts [184].

### 4.8. Blackberry

Blackberries (*Rubus fruticosus* L.) belong to the family of Rosaceae, and are rich in poly phenolics such as ellagic acid, tannins, ellagitannins, quercetin, gallic acid, anthocyanins, and cyanidins that are best known for their high antioxidant action [203].

In HT-29 and HCT-116 cells, phenolic-rich blackberry extracts inhibited cell proliferation and induced apoptosis by internucleosomal DNA degradation at different concentrations [145]. Anthocyanin rich extracts from hull and crude blackberries showed significant antioxidant and antiproliferative activity in HT-29 cells [185] and inhibited 2,2′-Azobis (2-amidinopropane) dihydrochlorid induced oxidative damage and associated cytotoxicity in Caco-2 colon cancer cells [186].

Blackberries exhibited chemopreventive effects against chemically induced colon carcinogenesis in male Fisher 344 rats. Dietary administration of blackberry juice also significantly reduced the formation of AOM induced ACF in rats [150].

### 4.9. Blackcurrant

Blackcurrant fruit (*Ribes nigrum* L.; family: Grossulariceae) is commonly rich in phytonutrients, vitamin C and antioxidants. The antiproliferative effect of blackcurrant extracts has been reported on colon cancer HT-29 cells. The suppression of cancer cell proliferation was correlated with antioxidant capacity [142]. Blackcurrant press residue extracts rich in anthocyanins and polyphenols inhibited the proliferation of several colon cancer cells, including Caco-2, HT-29 and HCT-116. The extracts obtained from high temperature induced higher antiproliferative activity compared to lower temperature [187]. In HT-29 cells, the antiproliferative effect was induced by induction of apoptosis [187] and suppression of the p21WAF1 pathway [168]. In another study, blackcurrants showed anticancer activity after IVD and fecal fermentation in HT-29 and HT-115 cells through the inhibition of key stages of initiation, promotion and invasion [116].

### 4.10. Chokeberry

Chokeberries (*Aronia melanocarpa* L.) belong to the Rosaceae family. They have attracted substantial attention because of their high content of antioxidants and polyphenols (procyanidins, anthocyanins and phenolic acids) [76,204].

To mimic physiological conditions, Bermúdez-Soto and colleagues subjected chokeberry juice to *in vitro* gastric and pancreatic digestion. They found that exposure to chokeberry juice inhibited Caco-2 cell proliferation by causing G2/M cell cycle arrest [125]. It also changed the expression of some genes associated to colorectal cancer, such as carcinoembryonic antigen-related cell adhesion molecule 1 (CEACAM1) gene which has an important regulatory role on cell proliferation [125]. Additionally, anthocyanin rich chokeberry extracts induced chemopreventive activity in HT-29 cells by inhibiting the proliferative activity [153,154]. In another study, chokeberry extracts reduced cell cycle progression mainly by blocking G1/G0 and G2/M phases, which coincided with increased expression of p21^WAF^ and p27^kip1^ genes and decreased expression of cyclin A and B genes. Furthermore, COX-2 gene expression was also observed in HT-29 cells treated with chokeberry extract [188]. Finally, anthocyanin-rich extracts of chokeberry fruit significantly inhibited colonic ACF formation by decreasing colonic cell proliferation in male rats treated with a colon carcinogen, AOM but did not change other biomarkers [158].

### 4.11. Cloudberry

Cloudberry (*Rubus chamaemorus* L., family: Rosaceae) seeds and pulp are used as ellagitannin sources with a high level of ellagic acid that exhibits potent anticarcinogen, antimutagen and antioxidant activity [78,79,205].

Polyphenol rich extracts from cloudberries showed significant antiproliferative activity in Caco-2 cells [162]. In HT-29 cells, cloudberry extracts inhibited cellular growth by increasing the expression of p21^WAF1^ pathway and induced apoptosis by increasing Bax mRNA expression [168].

Dietary administration of freeze dried cloudberries significantly reduced tumor number and size of intestinal adenomas in Min/+ mice. In large adenomas, cloudberries decreased levels of nuclear β-catenin and cyclin D1. In addition, affymetrix microarrays exposed changes in genes involved in colon carcinogenesis, including the decreased expression of the adenosine deaminase, ecto-5’–nucleotidase and PGE2 receptor subtype EP4 [171].

### 4.12. Seabuckthorn

Seabuckthorn (*Hippophae rhamnoides* L., family: Elaeagnaceae) is a high-altitude medicinal plant with a large number of nutrients, phytochemicals, and bioactive substances like vitamin C [189].

Polyphenol rich extracts from seabuckthorn induced antiproliferative activity in HT-29 cells. Olsson *et al.*, suggested that the inhibition of cancer cell proliferation was correlated with vitamin C and carotenoid levels of seabuckthorn extracts [142]. A recent study has shown that isorhamnetin, a flavonoid isolated from seabuckthorn, suppressed HT-29, HCT-116 and SW480 cells proliferation [189]. Mechanistic studies revealed that the antiproliferative activity of seabuckthorn was mediated by arresting cell cycle at the G2/M phase and inhibiting the PI3K-Akt-mTOR signaling pathway [189].

Seabuckthorn seed oil also showed an *in vivo* potential role in protecting the colon tissue of rats from the 2-amino-1-methyl-6-phenylimidazo [4,5-b] pyridine (PhIP) induced oxidative damage. They found that PhIP significantly induced oxidative stress, activated immediate early genes c-fos and c-jun, and inhibited tumor suppressor genes p16 and Rb. On the other hand, seabuckthron seed oil significantly improved superoxide dismutase, catalase and glutathione peroxidase activities and reduced the malondialdehyde, protein carbonyl and DNA-protein cross-links levels in rat colons in the presence of PhIP [190]. Furthermore, seabuckthorn seed oil normalized abnormal expression of c-fos, c-jun, p16 and Rb mRNA genes [190].

### 4.13. Lingonberry

Lingonberries (*Vaccinium vitis-idaea* L., family: Ericaceae) present a complex polyphenolic profile consisting principally of a mixture of flavan-3-ols and proanthocyanidins [206] and exhibit a high antioxidant capacity [83,84,101].

The anthocyanin fraction of lingonberry extracts decreased the proliferation of colon cancer HT-29 cells in a concentration-dependent manner [142]. Recently, Wu *et al.*, reported that anthocyanin rich lingonberry extracts suppressed HT-29 cells growth by increasing expression of the p21^WAF1^ pathway [168]. Furthermore, McDougall and colleagues reported that lingonberry extracts exerted an antiproliferative effect against human colon cancer CaCo-2 cells, and the extracts were generally more sensitive at low concentrations but conversely less sensitive at higher concentrations [162].

Lingonberries exhibited *in vivo* chemopreventive properties by inhibiting adenoma formation in rat colon. Diet containing freeze dried lingonberries significantly reduced tumor number and tumor size in ApcMin/+ mouse colon. Cyclin D1 levels also decreased in large adenomas after feeding mice with lingonberries [171].

### 4.14. Barberry

Barberries (*Berberis vulgaris* L., family: Berberidaceae) have a long history of medicinal use for their multiple pharmacological and therapeutic effects. Various parts of this plant including its root, bark, leaf and fruit have been studied for its natural antioxidant and phenolic compounds [207,208].

Berberine, an isoquinoline alkaloid found in barberries, has been identified as a potent anticancer compound. It was reported that berberine caused inhibition of colon cancer SW480 cells growth by arresting cell cycle at G2/M phase, which was accompanied by induction of p21 expression. Berberine induced intrinsic pathway of apoptosis by loss of mitochondrial membrane potential, release of cytochrome-c to cytosol, induction of Bcl-2 family proteins, caspases and cleavage PARP [191]. Berberine also suppressed the expression of inflammation markers, NFκB and COX-2, suggesting its ability to inhibit inflammation. Furthermore, berberine inhibited caspase-8 mediated angiogenesis, as confirmed through the expression of TNF related apoptosis-inducing ligand, vascular endothelial growth factor (VEGF) and survivin [191].

### 4.15. Açai Berry

The açai berry (*Euterpe oleracea* Mart., family: Arecaceae) is a good source of phytochemicals like other berries. It is rich in anthocyanins, proanthocyanidins, other flavonoids and lignans .The pulp of this berry has been extensively studied for its antioxidant and anti-inflammatory activities [209].

Polyphenolic extracts of the açai berry showed anti-inflammatory and cytotoxic activities in colon cancer cells. They preferentially inhibited the growth of SW480 and HT-29 cells with no toxicity in nonmalignant CCD-18Co colon fibroblast cells [192]. Antiproliferative activity of açai berry extracts was accompanied by (i) reduction of H_2_O_2_ induced ROS generation; (ii) down-regulation of NF-κB and intracellular adhesion molecule-1 and vascular cell adhesion molecule-1. The polyphenolic extracts of açai berries also downregulated prooncogenic specificity proteins targets Bcl-2, VEGF, and surviving [192]. In addition, activation of mitochondrial proapoptotic pathway, involving increase of cytochrome c, cleavage of caspase-3, and decrease of PARP-1, was also observed in SW480 cells after treatment with açai berry extracts [192].

Recently, spray-dried açai powder was used for the prevention of early and late colon carcinogen in male Wistar rats. This berry powder significantly reduced the number of aberrant crypts, invasive tumors and tumor multiplicity. Additionally, reduction in tumor Ki-67 cell proliferation and net growth index was also noticed in the açai berry fed group [193].

### 4.16. Gogi Berry

Goji berries (*Lycium barbarum* L., family: Solanaceae) are well known in traditional herbal medicine. At present, they are used as a functional food with highly advantageous nutritive and antioxidant properties [210]. They are rich in Lycium barbarum polysaccharides (LBP) [211]. LBP treatment inhibited human colon cancer SW480 and Caco-2 cells growth by interruption of the cellcycle at the G0/G1 phase and reduction of cyclin D, cyclin E, and CDK2 espression [194].

### 4.17. Silverberry

The silverberry (*Elaeagnus* sp., family: Elaeagnaceae) is known as nutraceutical plant, which is used for both food and medicine. Lee and coworkers investigated the potentiality of silverberry against colon cancer. The extracts from seed and flesh of cherry silverberries induced anti-inflammatory and anti-proliferation properties in HT-29 cells by reducing COX-2 expression and induced apoptosis by decreasing phosphorylated Akt expression [195].

### 4.18. White Currant

The white currant (*Ribes* x *pallidum*, family: Grossulariaceae) is an interesting berry, containing low levels of phenolics, whereas proanthocyanidins and phenolic acids are the predominant phenolic compounds [90].White currant is effective in preventing cancer initiation and progression in Min mouse. It reduced the number and size of adenomas in the small intestine and in the colon, and in both places the area of adenomatous tissue did not increase. The chemopreventive effect of white currants was mediated by the reduction of nuclear β-catenin and NF-κB levels in Min mice adenomas [196].

### 4.19. Arctic Bramble

Arctic brambles (*Rubus arcticum* L., family: Rosaceae) are considered the most valuable berry plant of the genus *Rubus* because of their fine aroma and flavour. The major class of phenolic compounds in arctic brambles is represented by hydrolyzable tannins (gallo- and ellagitannins) and anthocyanins [78].

Polyphenol-rich extracts, especially ET enriched fractions, were found to be effective antiproliferative agents against human colon cancer Caco-2 cells. They were generally more sensitive at low concentrations but conversely less sensitive at higher concentrations [162].

### 4.20. Elderberry

The elderberry (*Sambucus nigra* L., family: Adoxaceae) has been used both as food and medicine since ancient times. It is rich in polyphenols and anthocyanins.

Anthocyanin rich elderberry extracts have been reported to induce antiproliferative activity in colon cancer HT-29 cells [153].

### 4.21. Jamun Berry

Jamun berries (*Syzygium cumini* L. Skeels, family: Myrtaceae) have traditionally been popular in the field of herbal medicine. The pharmacological activities are mainly attributed due to the presence of different flavonoids and alkaloids [212].

In human 293T cell line, ET rich jamun berry extracts produced colonic metabolities UAs which have potential against colon carcinogenesis. The anticarcinogenic effect of the jamun berry is mediated by inhibition of the canonical Wnt signaling pathway [166].

### 4.22. Rosehip

Rosehips (*Rosa villosa* L., family: Rosaceae) have a high content of vitamin C, carotenoids and phenolics [213].

Polyphenol rich extracts from rosehips inhibited HT-29 cell proliferation in a concentration-dependent manner, and antiproliferative activity was correlated with high levels of carotenoids and vitamin C [136] and flavonoids fraction [93].

### 4.23. Emblic

Emblic fruit (*Phyllanthus emblica* L., family: Phyllanthaceae) is commonly known as Indian Gooseberry. It is considered as a potent functional food due to its numerous pharmacological applications [214]. Hydrolyzable tannins and flavonoids are the major bioactive components of this fruit [215]. It has been reported that emblic extract showed antiproliferative activity in HT-29 cells [197]. In addition, emblic water extract inhibited genomic damage and cell death in human colon cancer COLO320 cells by several mechanisms. Emblic extracts induced a significant decrease in necrosis and nuclear division index as well as a marked increase in the frequency of chromosomal instability in a dose- and time-dependent manner [198]. Emblic extracts also significantly increased apoptosis, and there was a significant correlation of apoptosis with chromosomal instability [198].

## 5. Biological Activities of Berries Against colon Cancer: Human Studies

Individual case studies of successful treatment of colon cancer with different fruits (including berry) and vegetables have been reported for chemoprevention. A limited number of clinical investigations are available regarding the effect of various berry formulations on colon cancer (Table 4).

An anthocyanin-rich standardized bilberry extract, mirtocyan, showed chemopreventive efficacy. Twenty-five CRC patients were selected (primary tumor or liver metastases) and were given a certain amount of mirtocyan (from 1.4 to 5.6 g/day) for 7 days before surgery. Mirtocyan metabolites were identified in plasma, colorectal tissue, and urine, but not in the liver [216]. As a result, proliferation of tumor tissue was decreased by 7% compared with pre-intervention values. The low dose caused a small but non-significant reduction in circulating insulin-like growth factor (IGF)-1 concentrations [216].

In Phase I pilot study, Wang *et al.*, found that black raspberries effectively modulated both genetic and epigenetic biomarkers in tissues from CRC patients. Before and after oral consumption of black raspberry powder (60 g/day) for 19 weeks, biopsies of adjacent normal tissues and colorectal adenocarcinomas were collected from 20 patients [217]. Colon and rectal biopsies tissues showed that berries upstream demethylated tumor suppressor genes (SFRP2, SFRP5and WIF1) and PAX6a, a developmental regulatory gene, only in patients who received the berry treatment for an average of 4 weeks [217]. Black raspberries also protectively modulated the expression of genes associated with Wnt pathway (β-catenin, E-cadherin), cell proliferation, apoptosis, and angiogenesis [217]. In another study, freeze dried black raspberries attenuated neoplastic changes in 24 colorectal cancer patients who drank slurry of black raspberry powder (20 g in 100 mL drinking water) 3 times/day for 1–9 weeks. Before and during berry treatment, plasma and biopsy samples of colorectal adenocarcinoma and adjacent normal appearing tissues were taken. Patients who received the berry products for more than 10 days showed an increase in plasma concentration of granulocyte macrophage colony stimulating factor (GM-CSF), and decrease in IL-8. These changes also interacted with beneficial changes in markers of proliferation and apoptosis observed in colorectal tissue collected within the same week. The authors found the plasma concentrations of GM-CSF and IL-8 may serve as non-invasive indicators to monitor tissue response to berry-based interventions for CRC [218]. Wang and co-researchers suggested that black raspberries might degenerate rectal polyps in patients with FAP [219]. 14 FAP patients were treated with black raspberries, (orally and with suppositories inserted into the rectum) daily for 9 months. Oral supplementation did not provide additional benefits to the patients, but black raspberry suppositories significantly decreased Ki-67 levels, DNMT1, and p16 promoter methylation, in rectal polyps [219].

Blackcurrant powder reduced the activity of some colon cancer markers by acting as prebiotic agents. Consumption of first leaf (FL) (composed of blackcurrant extract powder, lactoferrin and lutein) and Cassis Anthomix 30 (CAM30; blackcurrant extract powder) significantly increased the number of beneficial bacteria, lactobacilli and bifidobacteria in the gut, whereas the population sizes of *Clostridium* spp. and *Bacteroides* spp. decreased significantly. In addition, consumption of FL and CAM30 reduced the activity of β-glucuronidase (bacterial enzyme that increases risk for colorectal cancer) and significantly decreased the fecal pH [220].

**Table 4:** Human intervention studies on colon cancer using fresh or processed berry fruits.

Fresh or Processed Berry	Study Subjects	Duration and Dose/Intervention	Key/Major Findings	References
Anthocyanin-rich standardized bilberry extract, mirtocyan	25 colorectal cancer patients	0.5–2.0 g/day for 7 days before surgery	- Prevents proliferation of tumor tissue.	[216]
Black raspberry power	20 colorectral cancer patients	60 g/day of black raspberry orally for 1 to 9 weeks	- Upregulates tumor suppressor gene. - Modulates expression of genes associated with Wnt pathway, proliferation, apoptosis and angiogenesis.	[217]
	24 colorectral cancer patients	20 g in 100 mL drinking water, 3 times/day for 1–9 weeks	- Induces chemoprevention by increasing markers of apoptosis in colorectal tissue. - Inhibits cell proliferation, and angiogenesis.	[218]
Black raspberry	14 patients with FAP	Oral treatment containing 60 g black raspberr/day, and suppositories containing 720 mg black raspberry/day for 9 months.	- Decreases cellular proliferation, DNMT1 protein expression, and p16 promoter methylation in adenomas.	[219]
FL and CAM30 prepared from blackcurrant extract	30 healthy volunteers (Aged 20–60 years)	Both product contain 672 mg blackcurrant power	- Expresses anticancer activity by decreasing the activity of the bacterial β-glucuronidase enzyme and lowering the fecal pH.	[220]

## 6. Conclusions

Berry fruits are rich in bioactive constituents, including flavonoids, anthocyanins, phenolic acids, stilbenes, and tannins, as well as nutritive compounds such as sugars, essential oils, carotenoids, vitamins and minerals. Numerous scientific studies provide ample evidence that bioactive compounds have the potential to prevent colon cancer risk. According to human studies, the chemopreventive effects of berries and berry products have focused mainly on black raspberries and bilberries. Some other berries such as arctic brambles, jamunberries, rosehips and emblic fruit are rich in bioactive compounds but very few sporadic efforts (only *in vitro*) have been made regarding their effects on colon cancer. *In vivo* studies need to be done on those berries.

Berry polyphenols and other bioactive compounds show anticancer effects on colon cancer thanks to their ability to influence carcinogen metabolism, scavenge free radicals and reduce oxidative damage to DNA. They activate several signaling pathways, including NF-κB, Wnt/β-catenin, PI3K/AKT/PKB/mTOR, and ERK/MAPK and regulate major cellular processes, including inflammation, proliferation and angiogenesis. Few studies show that berries constitute and also induce anti-metastasis activity by inhibiting key features of cancer development (Figure 1). These may provide clues for the development of novel agents that could be useful in cancer chemoprevention or chemotherapy. The microflora in colon are assuredly capable of metabolizing bioactive compounds of berries, producing metabolites that expose chemopreventive activity. Further work is needed to design *in vitro* and *in vivo* studies that better reflect the colonic environment. In addition, much work needs to be done on optimizing the bioavailability of berry polyphenols and determining their pharmacological applications. Thus, the association of identified and unidentified compounds in these fruits and their influence on colon cancer in targeted populations and patients makes this a very promising approach for the prevention and treatment of colon cancer.
